# Willingness to Use Long-acting Injectable Pre-exposure Prophylaxis Among Key Populations at a Large Hiv Prevention Clinic in Kampala, Uganda: a Cross-sectional Study

**DOI:** 10.21203/rs.3.rs-4719964/v1

**Published:** 2024-08-05

**Authors:** Jonathan Derrick Lukubuya, Elizabeth B. Katana, Micheal Baguma, Andrew Kaguta, Winnie Nambatya, Peter Kyambadde, Timothy R. Muwonge, Andrew Mujugira, Eva Agnes Laker Odongpiny

**Affiliations:** School of Pharmacy, College of Health Sciences, Makerere University; Uganda National Institute of Public Health, Ministry of Health; School of Pharmacy, College of Health Sciences, Makerere University; School of Pharmacy, College of Health Sciences, Makerere University; School of Pharmacy, College of Health Sciences, Makerere University; Most At Risk Populations Initiative (MARPI), Mulago National Referral Hospital; The Infectious Diseases Institute, College of Health Sciences, Makerere University; The Infectious Diseases Institute, College of Health Sciences, Makerere University; The Infectious Diseases Institute, College of Health Sciences, Makerere University

**Keywords:** HIV, Long-acting injectable Pre-exposure Prophylaxis (LAI-PrEP), Key populations, Female sex workers, People who inject drugs, Men who have sex with men, Truck drivers

## Abstract

**Background.:**

Long-acting injectable (LAI)-PrEP provides better protection against HIV compared to oral PrEP, which requires taking a daily pill. Our study aimed to assess knowledge about oral and LAI-PrEP and identify factors associated with willingness to use LAI-PrEP among key populations (KP) in Uganda.

**Methods.:**

We conducted a cross-sectional study at the Most at Risk Populations Initiative (MARPI) clinic between November and December 2021. Participants were recruited through convenience sampling and interviewed using a structured questionnaire by trained interviewers. Participants were categorised into three groups based on their oral PrEP use: those who had not yet initiated PrEP, those who had discontinued oral PrEP, and those currently on oral PrEP. Modified Poisson regression analysis was performed to determine factors associated with the participant’s willingness to use LAI-PrEP. Data was analysed using STATA 14 software.

**Results.:**

Of the 234 participants, 135 (56.7%) were female, 82.5% knew about LAI-PrEP, and 67.5% were willing to use it. The mean age was 28.7 years (standard deviation [SD] 5.8). Willingness to use LAI-PrEP was less likely among divorced, widowed, or separated individuals than those in relationships (adjusted prevalence ratio [aPR] 0.65, 95% CI: 0.43–0.98). Relative to current oral PrEP users, willingness to use LAI-PrEP was similar among those who discontinued oral PrEP (aPR 1.39, 95% CI: 0.92–2.11) and those who had not yet initiated PrEP but were at risk for HIV (aPR 1.26, 95% CI: 0.83–1.89).

**Conclusions.:**

In this cross-sectional analysis of diverse members of key populations in Uganda, previous or non-use of oral PrEP was not associated with willingness to use LAI-PrEP relative to current users. Future studies should investigate effective methods for promoting the uptake of long-acting PrEP formulations among populations at high risk of HIV acquisition.

## BACKGROUND

Despite significant advancements in antiretroviral therapy and a decrease in mortality rates, HIV remains a significant global health issue after more than four decades. There are currently 37.7 million people living with HIV worldwide (range 30.2–45.1 million), and in 2020, there were 1.5 million new infections globally (1–3). Most people living with HIV (PLHIV) (71%) reside in sub-Saharan Africa, where over three-quarters of all deaths related to HIV occur, and approximately two-thirds of the estimated 4,000 daily new infections worldwide take place (1, 4). In Uganda, the adult HIV prevalence was 5.1%, with an estimated 52,000 new infections in 2022 (5). Most HIV transmissions in Uganda (70%) occur among key populations (KP), specifically sex workers (SW), people who inject drugs (PWID), truck drivers (TD), fisher folk (FF), and men who have sex with men (MSM) (4, 6). In these populations, the prevalence of HIV is 31.3%, 14.9–35.0%, 16.0%, and 12.7% among SW, FF, PWID, and MSM, respectively (7, 8).

Pre-exposure prophylaxis (PrEP) for HIV is a highly effective strategy in preventing HIV acquisition. The World Health Organization (WHO) recommends PrEP as an additional preventative measure for individuals at high risk of acquiring HIV in conjunction with other combination HIV prevention approaches (9). Although there has been a substantial uptake of PrEP in HIV prevention programs, persistence remains a challenge. Research conducted in Kenya, South Africa, and the United States revealed that over half of individuals who began taking oral PrEP discontinued use within the first six months (10–12). Other work conducted in Uganda showed low PrEP uptake (30.6%), high discontinuation (67.9%), and poor adherence, where 38.8% of adolescent girls and young women (AGYW) had detectable tenofovir (> 10 μg/L), and only 6.1% had protective tenofovir levels (> 40 μg/L) (13).

Long-acting injectable (LAI)-PrEP, formulated for multi-month use, was developed to overcome some of the challenges associated with oral PrEP. It offers numerous benefits, including being discreet and less stigmatising, making it a potential alternative to oral PrEP (14). LAI-PrEP was safe and preferred over oral PrEP in the HPTN 083 trial, resulting in 66% fewer HIV infections among cisgender men and MSM (15). While LAI-PrEP has various advantages compared to oral PrEP, its acceptability among KPs in Uganda is not well described. This study aimed to assess knowledge of LAI-PrEP and identify factors related to its usage among KPs, including those who had discontinued oral PrEP.

## METHODS

### Study design and setting

We conducted a cross-sectional study at the Most at Risk Populations Initiative (MARPI) clinic between November and December 2021. MARPI is a prominent HIV prevention clinic located in Mulago Hospital, Kampala, which provides essential services to the KP community, including counselling and testing, family planning, condom distribution, sexually transmitted infection (STI) screening, and other related services. The target populations of MARPI are female sex workers (FSW), TD, MSM, FF, transgender individuals, and bar attendants (16). We categorised potential LAI-PrEP beneficiaries into three groups: (i) eligible but had not yet initiated PrEP (naïve/not PrEP exposed), (ii) discontinued oral PrEP use, and (iii) currently taking oral PrEP.

### Recruitment and sampling

We used data extracted from MARPI PrEP registers to identify and recruit individuals ≥ 18 years old who were invited to visit the MARPI clinic. Through convenience sampling, 234 participants were selected for potential inclusion in the study. Informed consent was obtained before face-to-face interviews were conducted at the MARPI clinic. The desired sample size was 252, but only 234 participants were included due to the unwillingness of 18 selected individuals to be interviewed, who were not replaced.

### Data collection

We used an interviewer-administered semi-structured questionnaire to collect data for the study. Before implementation, the questionnaire was pretested with ten community volunteers who were identified as being at risk for HIV. Trained research assistants contacted and administered the questionnaire to eligible participants. The primary objective of this study was to determine the level of willingness among KPs to use LAI-PrEP, recorded on a binary scale with responses of either “Yes” or “Not sure/No.” Additionally, we documented reasons for declining LAI-PrEP, circumstances in which they would be more inclined to receive it, and their preferred location for obtaining it.

### Statistical analysis

The primary outcome was willingness to use LAI-PrEP. Participant characteristics were summarised using descriptive statistics. Modified Poisson regression was used to determine factors associated with willingness to use LAI-PrEP when it became available and to estimate prevalence ratios (PR) and corresponding 95% confidence intervals (CI). Both unadjusted and adjusted modelling were performed, with the adjusted model including only significant factors (p < 0.05) from the unadjusted model. Covariates in the model included age, KP category, marital status, race, gender, education, occupation, number of sex partners in the previous month, history of unprotected sex in the past 3 months, use of illicit drugs before sex in the previous 6 months, previous STI diagnosis, condom use, knowledge of PrEP and HIV risk category. Logistic regression was not performed because the primary outcome’s high prevalence (> 10%) could lead to biased odds ratio estimates. Data was analysed using STATA 14.

## RESULTS

### Socio-demographic characteristics

Of the 234 participants, 135 (56.7%) were female, with 115 (49.2%) of these women being SW. The mean age was 28.7 years (standard deviation [SD] 5.8). Most participants engaged in unprotected sexual activity within the last 3 months (189; 80.8%), reported having more than two sexual partners in the previous month (117; 75.6%), and expressed fear of acquiring HIV (158; 67.8%) (Table 1). Nearly half had been previously diagnosed with STIs (114; 48.9%). Seventy-nine (33.9%) frequently used illicit drugs before sex in the past 6 months and only 76 (32.5%) consistently used condoms. Most participants (193; 82.5%) were aware of LAI-PrEP, with the highest level of knowledge observed among FSW (88.7%). A total of 102 (43.7%) were currently taking oral PrEP, 75 (32.1%) were at risk but PrEP naïve, and 57 (24.4%) had discontinued oral PrEP. All participants knew about oral PrEP; 173 (73.9%) had heard about oral PrEP from health workers, 21 (15.7%) from a friend and 15 (11.2%) from social media.

### Willingness to use long-acting injectable PrEP

Two-thirds (154; 65.8%) were willing to use LAI-PrEP. Most participants (77.2%; 44/57) who had discontinued oral PrEP were willing to use LAI-PrEP, while 60.0% (45/75) who were at risk but PrEP naïve and 63.7% (65/102) who were using oral PrEP were willing to use LAI-PrEP. Most participants reported that they would be more interested in LAI-PrEP for the following reasons: 209 (89.3%) if they could get access to free sexual health care/monitoring while receiving LAI-PrEP, 167 (71.4%) if they could get free HIV testing, 148 (63.2%) if they did not have to go to their regular doctor to get LAI-PrEP, 122 (52.1%) if they could talk to someone and get support or counselling about their sex life, 117 (50.0%) if they could have access to one-on-one counselling and support around LAI-PrEP use, 85 (36.3%) if they could get text-based support for injectable PrEP use, and 92 (39.3%) if they could have group-based adherence support for injectable PrEP use. Reasons for not being willing to use LAI-PrEP included myths and misconceptions like “If I become HIV positive, the medicines may not work because I was on LAI-PrEP’’ (26; 44.8%) and LAI-PrEP putting themselves at increased risk of HIV (14; 24.1%) (Table 2).

### Factors associated with willingness to use LAI-PrEP

In the multivariate analysis, participants who were not currently in a relationship had a decreased likelihood of interest in LAI-PrEP than those who were single (adjusted prevalence ratio [APR] 0.65; 95% CI: 0.43–0.98; p = 0.04). Compared with current PrEP users, there was no significant difference in willingness to use LAI-PrEP between those who discontinued oral PrEP (APR 1.39; 95% CI: 0.92–2.11; p = 0.12) or those who had never used PrEP (APR 1.26; 95% CI: 0.83–1.89; p = 0.28) (Table 3).

## DISCUSSION

In this cross-sectional study, we explored the willingness to use LAI-PrEP among KPs in Kampala when categorised into different PrEP use groups (i.e., those taking oral PrEP, those eligible but PrEP naïve, and those who discontinued oral PrEP). Of the 234 members of key populations in Uganda, two-thirds were willing to use LAI-PrEP. Those who were sexually active showed a greater interest in injectable PrEP compared to those not currently in a relationship. There was no significant difference in willingness to use LAI-PrEP between former oral PrEP users and individuals who were PrEP naive.

Our findings are consistent with previous research conducted in Nigeria and Thailand, which found a high willingness among KPs to use LAI-PrEP. In Nigeria, a study focusing on MSM reported an 88% willingness to use LAI-PrEP, while another study in Bangkok focusing on people who inject drugs reported a 73.5% willingness (24, 29). A separate study among KPs also found a strong interest in using LAI-PrEP among adolescents, transgender women, and FSW (17). However, our study found a lower level of willingness to use LAI-PrEP among MSM compared to a similar study conducted in the United States (80%), which may be attributed to the limited awareness of LAI-PrEP in Uganda (18). Additionally, a study among women in Zimbabwe, South Africa, and the United States showed that the majority (> 75%) rated injectable PrEP as acceptable (19).

Willingness to use LAI-PrEP varied across the study groups, with the highest reported among individuals who had previously discontinued oral PrEP and the lowest observed among those currently taking oral PrEP. These findings are consistent with the ÉCLAIR study, which also found a high level of willingness (79%) among participants to continue utilising LAI-PrEP, with an even higher percentage (87%) indicating they would recommend it to others (20). In comparison, a greater proportion of individuals who had discontinued oral PrEP expressed a willingness to switch to LAI-PrEP versus those currently taking oral PrEP or individuals within the eligible PrEP-naïve key populations. This difference is likely due to challenges in accessing necessary healthcare services or other challenges with adherence to oral formulations despite the need for PrEP (21). Among specific key population groups, willingness to switch to LAI-PrEP varied from ~ 60%−100%, higher than the 30.8%−66.7% reported in US-based studies of gay and bisexual men taking oral PrEP (21–23). Moreover, a study conducted in South Africa among heterosexual men found that 48% of participants favoured LAI-PrEP, while 33% and 20% opted for oral PrEP and condoms, respectively (18). Variations in knowledge levels may explain this disparity, as our study revealed high levels of knowledge regarding LAI-PrEP.

Our research revealed that individuals who were divorced, widowed, or separated had a decreased likelihood of being willing to use LAI-PrEP compared to those who were single. This finding is consistent with a study conducted in Uganda among adolescent boys and young men in Eastern Uganda, which identified a negative association between being unmarried and willingness to use LAI-PrEP (24). Another cohort study among FSWs in Tanzania also found that being married/cohabiting or separated/divorced/widowed was independently associated with the use of oral PrEP, which may also apply to LAI-PrEP (25). However, we did not observe significant differences in willingness to use LAI-PrEP among participants who discontinued oral PrEP and those who were PrEP naive relative to oral PrEP users. In contrast, studies involving gay and bisexual men have shown a higher level of willingness to use LAI-PrEP among oral PrEP users (26). Our research revealed that among those who were not willing not to take LAI-PrEP, reasons included myths/ misconceptions and potentially stigma-driven reasons. These will need to be addressed to effectively roll out a LAI-PreP programme. Others include preferences for taking tablets and that taking tablets served as a reminder to also take their other medications. However, other studies have shown that participants’ main concern regarding LAI-PrEP is its potential long-term side effects (27).

Uganda currently ranks third in Africa for the number of PrEP initiations, with a total of 599,786, behind South Africa (1,323,845) and Zambia (697,980) (28). Despite high PrEP uptake, only < 15% of those who initiate PrEP return for their first refill. Reasons for discontinuing oral PrEP include side effects, transport costs to the clinic, stigma, and the inconvenience of a daily pill. The introduction of LAI-PrEP) has the potential to significantly improve PrEP persistence. However, even though Uganda’s National Drug Authority approved the use of injectable cabotegravir (CAB-LA) as PrEP in February 2024, access is limited to demonstration projects. The slow global rollout of LA-CAB has hindered its population-level impact on reducing HIV incidence. Delays in scaling up CAB-LA in resource-limited settings can be attributed to slow regulatory approvals, production challenges, supply chain limitations, and its high cost, ranging from $170-$240 per year, according to estimates by PEPFAR (23, 29, 30).

### Study limitations and strengths

The limitations of our study include its cross-sectional design, lack of access to LAI-PrEP in Uganda during the study period, and the potential for recall and social desirability bias. Furthermore, due to limitations caused by the COVID-19 lockdown, key populations had restricted access to healthcare services, which could have impacted their availability to participate in the study. Additionally, our sample was limited to an urban population and may not fully represent all KP in the country. However, despite these limitations, our study on the willingness of KP to use LAI-PrEP can provide valuable insights for future planning and implementation when LAI-PrEP becomes available in Uganda.

## CONCLUSIONS

In conclusion, our study showed that most key population members in our sample were aware of LAI-PrEP, and approximately two-thirds were willing to use it. However, those who were divorced, widowed, or separated had lower likelihood of being willing to use LAI-PrEP compared to their single counterparts. Considering the potential adherence benefits of LAI-PrEP, further research is needed to assess effective demand-creation strategies for KPs to enhance acceptability and willingness to use this effective HIV prevention method.

## Figures and Tables

**Table 1:** Sociodemographic characteristics

Characteristic	Taking oral PrEP (n = 102)	Eligible but PrEP naïve (n = 75)	Discontinued oral PrEP (n = 57)	Total (n = 234)
	N (%)			
**Age (years)**				
University/post-university	43 (42.2)	0	0	43 (18.5)
None	2 (2.0)	6 (8.0)	9 (16.1)	17 (7.3)
**Marital Status**				
Divorced/separated	23 (22.6)	42 (56.0)	21 (36.8)	86 (36.8)
Single	16 (15.7)	21 (28.0)	24 (42.1)	61 (26.1)
Cohabiting	27 (26.5)	2 (2.7)	7 (12.3)	36 (15.4)
Married (Polygamous)	31 (30.4)	10 (13.3)	3 (5.3)	44 (18.8)
Widowed	5 (4.9)	0	2 (3.5)	7 (3.0)
**Occupation** **				
Labourer (Semi skilled)	25 (25.3)	26 (34.7)	10 (17.5)	61 (26.4)
Farming	23 (23.2)	0	0	23 (10.0)
Traders/sales	18 (18.2)	0	0	18 (7.8)
Student	14 (14.1)	3 (4.0)	5 (8.7)	22 (9.5)
Unemployed	0	0	13 (22.8)	13 (5.6)
Professional	9 (9.1)	0	0	9 (3.9)
Housewife	3 (3.0)	0	0	3 (1.3)
Other	7 (7.1)	46 (61.3)	29 (59.9)	82 (35.5)
**Sex Partners (previous month)**				
More than two	47 (46.1)	75 (100)	55 (96.5)	177 (75.6)
Two	29 (28.4)	0	2 (3.5)	31 (13.3)
One	23 (22.6)	0	0	23 (9.8)
None	3 (2.9)	0	0	3 (1.3)
**Had unprotected sex in the last 3 months**				
Yes	61 (59.8)	75 (100)	53 (93.0)	189 (80.8)
No	22 (21.6)	0	4 (7.0)	26 (11.1)
Not sure	19 (18.6)	0	0	19 (8.1)
**Used illicit drug before sex in previous 6 months** *				
All the time/very frequently	41 (40.2)	15 (20.3)	23 (40.4)	79 (33.9)
Sometimes	0	0	34 (59.7)	34 (14.6)
No	49 (48.0)	32 (43.2)	0	81 (34.8)
Not sure	12 (11.8)	27 (36.5)	0	39 (16.7)
**Previous STI diagnosis** *				
Yes	37 (36.3)	64 (85.3)	13 (23.2)	114 (48.9)
No	52 (51.0)	11 (14.7)	38 (67.9)	101 (43.4)
Not sure	13 (12.7)	0	5 (8.9)	18 (7.7)
**Use condoms**				
All the time/very frequently	38 (37.3)	53(70.0)	51 (89.5)	142 (60.7)
Sometimes	51 (50.0)	22 (29.3)	0	73 (31.2)
No	13 (12.8)	0	6 (10.5)	19 (8.1)

* 1 missing,

** 2 missing observations

**Table 2:** Willingness to use long-acting injectable PrEP

Characteristic	Taking oral PrEP (n = 102)	Eligible but PrEP naïve (n = 75)	Discontinued oral PrEP (n = 57)	Total (n = 234)
	N (%)			
**Willingness to use long-acting injectable PrEP** *				
Yes (willing)	65 (63.7)	45 (60.0)	44 (77.2)	154 (65.8)
No (not willing)	34 (33.3)	11 (14.7)	13 (22.8)	58 (24.8)
Not sure/Maybe	3 (2.9)	13 (17.3)	0	16 (6.8)
**If not, why? n = 58**	**n = 34**	**n = 11**	**n = 13**	
If I become HIV positive, the medicines may not work because I was on LAI-PrEP	26 (76.5)			26 (44.8)
Prefer taking tablets		11 (100)	13 (100)	24 (41.4)
Tablets will remind me of taking my other medicines		11 (100)	13 (100)	24 (41.4)
People will think I am HIV positive	23 (67.6)			23 (39.7)
Don’t want to talk to the doctor about my sex life	18 (52.9)			18 (31.0)
Because I will be putting myself at risk of HIV	14 (41.2)			14 (24.1)
May affect my other medicines	8 (23.5)			8 (13.8)
May not work	7 (20.6)			7 (12.1)
May have side effects	6 (17.6)			6 (10.3)
I fear Injections	3 (8.8)			3 (5.2)
Because I am not exposed to HIV				
My partner will see me.				
**I would be more interested in Injectable PrEP if I could get free HIV testing**				
Agree	39 (38.2)	72 (96.0)	56 (98.2)	167 (71.4)
Indifferent	56 (54.9)	2 (2.7)	1 (1.8)	59 (25.2)
Disagree	7 (6.9)			7 (3.0)
**Would be more interested in Injectable PrEP if I could get access to free sexual health care/monitoring while receiving Injectable PrEP**				
Agree	84 (82.4)	69 (92.0)	56 (98.2)	209 (89.3)
Indifferent	13 (12.7)	5 (6.7)	1(1.8)	19 (8.1)
Disagree	4 (3.9)			4 (1.7)
**I would be more interested in Injectable PrEP if I could have access to one-on-one counselling and support around Injectable PrEP use**				
Agree	34 (33.3)	40 (53.3)	43 (75.4)	117 (50.0)
Indifferent	65 (63.7)	26 (34.7)	12 (21.1)	103 (44.0)
Disagree	3 (2.9)	8 (10.7)	2 (3.5)	13 (5.5)
**I would be more interested in Injectable PrEP if I could get text-based support for Injectable PrEP use**				
Agree	61 (59.8)	20 (26.7)	4 (7.0)	85 (36.3)
Indifferent	28 (27.5)	28 (37.3)	29 (50.9)	85 (36.3)
Disagree	13 (12.7)	26 (34.7)	24 (42.1)	63 (26.9)
**I would be more interested in Injectable PrEP if I could talk to someone and get support or counselling about my sex life**				
Agree	49 (48.0)	70 (93.3)	3 (5.3)	122 (52.1)
Indifferent	38 (37.3)		28 (49.1)	66 (28.2)
Disagree	15 (14.7)	2 (2.7)	26 (45.6)	43 (18.4)
**I would be more interested in Injectable PrEP if I could have group-based adherence support for Injectable PrEP use**				
Agree	33 (32.4)	51 (68.0)	8 (14.0)	92 (39.3)
Disagree	25 (24.5)		37 (64.9)	62 (26.5)
Indifferent	43 (42.2)	21 (28.0)	12 (21.1)	76 (32.5)
**If you were to use PrEP (oral/injectable) where would you prefer to get this from?** **				
PrEP Clinic	80 (78.4)	42 (56.0)	23 (40.4)	145 (61.9)
Home	8 (7.8)	20 (26.7)	30 (52.6)	58 (24.8)
Community Pharmacy	8 (7.8)	10 (13.3)		18 (7.1)
Office	1 (0.9)	2 (2.7)		3 (1.3)
Other	5 (4.9)		4 (7.0)	9 (3.8)
Willingness to use LAI-PrEP by key population category*				
Key population	Not willing n = 58	Not sure/maybe n = 16	Willing n = 154	Total
Female sex worker	31 (26.9)	11 (9.6)	69 (60.0)	115 (49.2)
Truck driver	12 (30.0)	4 (10.0)	24 (60.0)	40 (17.1)
People who inject drugs (PWID)	5 (15.2)	1 (3.0)	27 (81.8)	33 (14.1)
MSMs	8 (28.6)	0	18 (64.3)	28 (12.0)
None	0	0	10 (100)	10 (4.3)
Transgender	0	0	4 (100)	4 (1.7)
Others	2 (50.0)	0	2 (50.0)	4 (1.7)

* 6 missing values,

** 1 missing value

**Table 3:** Factors associated with willingness to use long-acting injectable PrEP

Variable, (n = 228)	Willing, n = 154	Not willing or not sure, n = 74	cPR (95% CI), P value	aPR (95% CI), P value
**Study group**				
Previously on oral PrEP but discontinued use	44 (77.2)	13 (22.8)	1.21 (0.83–1.77), 0.326	1.39 (0.92–2.11), 0.116
At risk but are naïve about PrEP	45 (65.2)	24 (34.8)	1.02 (0.70–1.50), 0.905	1.26 (0.83–1.89), 0.278
Currently on oral PrEP	65 (63.7)	37 (36.3)	1.00 (ref)	1.00 (ref)
**Age in years**				
18–30	97 (66.9)	48 (33.1)	1.00 (ref)	
31–40	46 (66.7)	23 (33.3)	0.99 (0.70–1.42), 0.985	
41–45	11 (78.6)	3 (21.4)	1.17 (0.63–2.19), 0.613	
**Gender**				
Male	69 (71.1)	28 (28.9)	1.09 (0.80–1.51), 0.570	
Female	85 (64.9)	46 (35.1)	1.00 (ref)	
**Key population**				
Transgender	4 (100)	0	1.61 (0.58–4.41), 0.355	
PWID	27 (81.8)	6 (18.2)	1.32 (0.84–2.05), 0.226	
MSMs	18 (69.2)	8 (30.8)	1.11 (0.66–1.87), 0.684	
FSW	69 (62.2)	42 (37.8)	1.00 (ref)	
Truckers	24 (60.0)	16 (40.0)	0.96 (0.61–1.53), 0.881	
None	10 (100)	0	1.61 (0.83–3.12), 0.160	
Others (fisher folk, bar attendants)	2 (50.0)	2 (50.0)	0.80 (0.19–3.28), 0.761	
**Education**				
University/graduate	34 (79.1)	9 (20.9)	1.15 (0.58–2.27), 0.687	
Secondary	57 (70.4)	24 (29.6)	1.02 (0.54–1.95), 0.944	
Primary	52 (59.8)	35 (40.2)	0.86 (0.45–1.66), 0.673	
None	11 (68.7)	5 (31.3)	1.00 (ref)	
**Marital Status**				
Married/Cohabiting	65 (82.3)	14 (17.7)	1.07 (0.74–1.56), 0.714	1.23 (0.81–1.86), 0.328
Single	46 (76.7)	14 (23.3)	1.00 (ref)	1.00 (ref)
Currently not in a relationship	43 (48.3)	46 (51.7)	0.63 (0.42–0.95), 0.029	0.65 (0.43–0.98), 0.042
**Occupation**				
Student/Unemployed/Housewife	28 (73.7)	10 (26.3)	3.32 (0.79–13.9), 0.101	
Traders/sales	13 (72.2)	5 (27.8)	3.25 (0.73–14.4), 0.121	
Farming /Labourer (Semi skilled)	59 (71.9)	23 (28.1)	3.24 (0.79–13.3), 0.102	
Professional	2 (22.2)	7 (77.8)	1.00 (ref)	
Others	49 (62.8)	29 (37.2)	2.83 (0.68–11.6), 0.150	
**Number of sex partners previous month**				
One	13 (56.5)	10 (43.5)	0.56 (0.16–1.98), 0.373	
Two	22 (71.0)	9 (29.0)	0.71 (0.21–2.37), 0.577	
More than two	116 (67.8)	55 (32.2)	0.68 (0.22–2.13), 0.507	
None	3 (100)	0	1.00 (ref)	
**Had unprotected sex in the last 3 months**				
Yes	124 (67.8)	59 (32.2)	1.10 (0.65–1.85), 0.717	
No	16 (61.5)	10 (38.5)	1.00 (ref)	
Not sure	14 (73.7)	5 (26.3)	1.19 (0.58–2.45), 0.623	
**Used illicit drug before sex in the previous 6 months**				
Yes/ Very frequently	61 (80.3)	15 (19.7)	1.0 0(ref)	
Sometimes	26 (76.5)	8 (23.5)	0.95 (0.60–1.51), 0.836	
No	50 (62.5)	30 (37.5)	0.78 (0.53–1.13), 0.190	
Not sure	16 (43.2)	21 (56.8)	0.54 (0.31–0.93), 0.028	
**Previously diagnosed with STI**				
Yes	69 (63.9)	39 (36.1)	1.00 (ref)	
No	73 (72.3)	28 (27.7)	1.13 (0.81–1.57), 0.463	
Not sure	11 (61.1)	7 (38.9)	0.96 (0.51–1.81), 0.891	
**Use condoms**				
All the time/ Very frequently	97 (70.3)	41 (29.7)	1.00 (ref)	
Sometimes	42 (59.2)	29 (40.9)	0.84 (0.58–1.21), 0.350	
No	15 (78.9)	4 (21.1)	1.12 (0.65–1.93), 0.675	
**Heard about LAI-PrEP**				
Yes	125 (66.8)	62 (33.2)	1.00 (ref)	
No	20 (71.4)	8 (28.6)	1.02 (0.66–1.71), 0.783	
Not sure	3 (50.0)	3 (50.0)	0.75 (0.24–2.35), 0.619	

cPR = Crude Prevalence Ratio, aPR = Adjusted Prevalence Ratio

## Data Availability

The de-identified patient data has been attached as a supplementary file (supplementary file 1).
